# Preclinical Effectiveness of Selective Inhibitor of IRS-1/2 NT157 in Osteosarcoma Cell Lines

**DOI:** 10.3389/fendo.2015.00074

**Published:** 2015-05-13

**Authors:** Cecilia Garofalo, Mariantonietta Capristo, Caterina Mancarella, Hadas Reunevi, Piero Picci, Katia Scotlandi

**Affiliations:** ^1^Experimental Oncology Laboratory, CRS Development of Biomolecular Therapies, Rizzoli Institute, Bologna, Italy; ^2^TyrNovo Ltd., Tel Aviv, Israel

**Keywords:** NT157, IRS-1, osteosarcoma, IGF system, sarcoma, chemotherapy

## Abstract

Osteosarcoma (OS) is the most common primary bone tumor in children and young adults. Several studies have confirmed the involvement of the insulin-like growth factor (IGF) system in the regulation of OS cell proliferation and differentiation as well as in the protection of cells from chemotherapy. Insulin receptor substrate (IRS)-1 is a critical mediator of IGF-1R signaling, and we recently reported that its overexpression in OS cells increases proliferation, migration, and metastasis both *in vitro* and *in vivo*. In this study, we evaluated the efficacy of NT157, a selective inhibitor of IRS-1/2, in a panel of OS cells. A strong dose-dependent inhibition of growth was observed in the MG-63, OS-19, and U-2OS OS cell lines, displaying IC_50_ values at sub-micromolar doses after 72 h of treatment. Exposure to NT157 elicited dose- and time-dependent decreases in IRS-1 levels. Moreover, a protein analysis showed that the degradation of IRS-1 inhibited the activation of principal downstream mediators of the IGF pathway. NT157 significantly affected the cells’ migratory ability, as confirmed by a wound-healing assay. The inhibitor induced cytostatic effects, as evidenced by G2/M cell cycle arrest, and did not affect apoptosis. Consequently, NT157 was combined with drugs used to treat OS in order to capitalize on its therapeutic potential. Simultaneous treatments were made in association with chemotherapeutic agents in a fixed ratio for 72 h and cell proliferation was determined by MTT assay. Synergistic or addictive effects with respect to single agents are expressed as the combination index. Significant synergistic effects were obtained with several targeted drugs, such as Everolimus, a mammalian target of rapamycin (mTOR) inhibitor, and NVP-BEZ235, a dual inhibitor of PI-3K/mTOR. Overall, these findings provide evidence for the effectiveness of a selected inhibitor of IRS-1/2 NT157 in OS cells, displaying a promising approach based on the targeting of IRS-1 combined with other therapies for the treatment of this pediatric solid tumor.

## Introduction

The activation of the insulin-like growth factor (IGF) system regulates several aspects of the malignant phenotype, including the development and progression of cancer and metastasis (1, 2). The IGF family consists of circulating ligands (IGF-1, IGF-II, insulin), at least four receptors [IGF-1R, M6P/IGF-IIR, insulin receptor (IR), and hybrid receptors], and six binding proteins (IGF-BPs). Although multiple proteins are involved in IGF signal transduction, the insulin receptor substrate (IRS) molecules are the primary family of adaptor proteins that function as intermediates of IR and IGF-IR (3). Six IRS proteins have been identified, but only IRS-1 and IRS-2 are widely expressed in normal tissue (4). A number of different physiologic pathways involved in both mitogenic and metabolic responses, such as steroids, hormones, cytokines, and integrins, can regulate IRS protein expression (5–8). Importantly, many of these effector-signaling pathways have been implicated in tumorigenesis and cancer progression. Although IRS-1 is most often related to tumor growth and proliferation, IRS-2 is most frequently associated with tumor motility and invasion. The tyrosine phosphorylation of IRS proteins induces the phosphorylation of mitogen-activated protein kinase (MAPK) and subsequently increases proliferation. This tyrosine phosphorylation also activates the p110 subunit of phosphatidylinositol 3-kinase (PI-3K), leading to a decreased apoptosis, and modulates the mammalian target of rapamycin (mTOR), resulting in translational adaptation (4). IRS-1 is constitutively activated in a variety of solid tumors, including breast cancers, leiomyomas, Wilms’ tumors, rhabdomyosarcomas, liposarcomas, leiomyosarcomas, and adrenal cortical carcinomas (9). In addition to their canonical function as cytosolic signal transduction molecules, IRS proteins can be shuttled to the nucleus and may contribute to the process of malignant transformation. In 3T3 fibroblasts, IGF-1R, the known oncogene SV40T and v-src caused IRS-1 nuclear translocation (10, 11), while other authors demonstrated growth in soft agar and tumorigenicity in nude mice induced by nuclear IRS-1, independently of the oncogene SV40 T-antigen (12). Similarly, the overexpression of nuclear IRS-1 was observed in 32D cells that express either human IGF-IR or SV40T (13). Once in the nucleus, IRS-1 can interact with transcription factors, such as β-catenin, ER-α, and the androgen receptor (AR), to modulate the promoter activity of several genes involved in malignant transformation (14–17).

Osteosarcoma (OS) is the most frequent primary malignant tumor of bone and predominately affects adolescents and young adults (18). The estimated incidence rate is two to three cases/million/year, and it is most common between 10 and 20 years of age. Although modern treatment protocols combine chemotherapy, surgery, and radiotherapy, the 5-year survival rate for non-metastasizing patients remains 60–70%, and this rate decreases to less than 30% for OS patients with metastases or relapsed disease (19, 20). Thus, novel clinical strategies are needed to improve the survival of these patients. IGFs are important regulator of growth and development in normal bone and play an important role in basal bone – cell proliferation. Because IGF-1 mediates the regulation of many growth hormone (GH) functions, the dysregulation of the GH/IGFs axis may favor the pathogenesis of OS (21). Although the expression levels of IGF-1 and IGF-II increased during normal osteoblastic terminal differentiation *in vitro*, the expression of IGF-1R progressively decreased (22), suggesting that the upregulation of the receptor but not the ligands is the aberrant condition in OS. Despite several *in vitro* and *in vivo* studies that have demonstrated the effectiveness of therapies against IGF-1R in enhancing the antitumor response in OS (23, 24), this targeted therapy has been of limited benefit to patients with recurrent or refractory bone and soft tissue sarcomas, including OS (25). This failure may be attributed to changes in other signaling pathways of downstream components that are independent of the expression of the receptor, such as Akt, mTOR, and IRS-1. Recently, Contaldo et al. (26) showed the influence of IRS-1 to sustain tumorigenicity of OS; indeed, *in vitro* and *in vivo* data showed that the overexpression of IRS-1 in OS increased tumor proliferation, motility capacity, and anchorage-independent growth compared with parental cells.

Thus, we herein investigated the preclinical efficacy of NT157, a novel small-molecule that specifically targets IRS protein, in OS cells. NT157 is a small-molecule inhibitor that induces Ser-phosphorylation and consequently the degradation of IRS-1 and IRS-2. The destruction of IRS-1/2 lead to the long-term dysregulation of IGF-1R signaling, which is responsible for the anti-proliferative activity in several cancers (27, 28). Here, we demonstrated that this compound inhibits tumor growth, cell cycle, and the motility of OS cells via the downregulation of IRS-1/IRS-2 proteins and their downstream mediators. In addition, *in vitro* combination studies were conducted to identify the best drug interaction between NT157 and therapies that are currently used to treat this tumor.

## Materials and Methods

### Drugs

The small-molecule inhibitor of IRS-1/2, NT157, was kindly provided by TyrNovo Ltd. (Israel) (27). Briefly, NT157 was dissolved in dimethyl sulfoxide (DMSO) to generate a 10-mM stock solution, which was stored at −80°C. Doxorubicin was purchased from Sigma (St. Louis, MO, USA), cisplatin was obtained from TEVA (Italy), and methotrexate was obtained from Pfizer (Italy). The signal transduction inhibitor that targets mTOR, Everolimus, was purchased from Sequoia Research Products (Pangbourne, UK). The PI-3K/mTOR dual inhibitor NVP-BEZ235 was kindly provided by Novartis (Basel, Switzerland). Working dilutions of all drugs were prepared immediately before use.

### Cell lines

The human OS cell lines U-2OS and MG-63 were provided by the American Type Culture Collection (ATCC). The IOR/OS-19 cell line was obtained from the Experimental Oncology Lab at the Rizzoli Institute (Bologna, Italy) and was previously described (29). All cell lines have recently been authenticated by STR analysis using genRESVR MPX-2 and genRESVR MPX-3 kits (serac, Bad Homburg, Germany). The following loci were verified: D16S539, D18S51, D19S433, D21S11, D2S1338, D3S1358, D5S818, D8S1179, FGA, SE33, TH01, and TPOX VWA. The last control was performed in November 2012. These cell lines were all tested for mycoplasma contamination every 3 months (last control, December 2014) using a MycoAlert mycoplasma detection set (Lonza, Nottingham, Ltd.). The cultures were maintained in Iscove’s modified Dulbecco’s medium (IMDM) supplemented with penicillin (20 U/ml), streptomycin (100 μg/ml) (Sigma), and 10% heat-inactivated FBS (Lonza) at 37°C in a humidified 5% CO_2_ atmosphere.

### Cell proliferation assay

To assess cellular growth, cells were seeded on 6-well plates (2 × 10^5^ cells/well) in IMDM plus 10% FBS. After 24 h, various concentration of NT157 (0.3–3 μM) were added, and the cells were exposed to this drug for up to 72 h. A dose–response proliferation was evaluated on harvested cells by Trypan Blue vital cell count.

For the combined treatment, cells were plated into 96-well plates (range 2,500–5,000 cells/well) and treated for 72 h with NT157 alone (control) or combined with fixed ratios of DXR (10:1), CDDP (1:10), MTX (100:1), NVP-BEZ235 (10:1), or Everolimus (1:10). Cell proliferation was determined with an MTT assay (Roche, Indianapolis, IN, USA) according to manufacturer’s instructions.

### Cell cycle analysis

After 48 h of treatment with NT157 alone (1–3 μM) or in combination with NVP-BEZ235 (50 nM), the cell cultures were incubated with 10 μmol/L bromodeoxyuridine (Sigma) for 1 h in a 5% CO_2_ atmosphere at 37°C. The harvested cells were fixed in 70% ethanol for 30 min. After DNA denaturation with 2 N HCl, 1 × 10^6^ cells were processed for indirect immunofluorescence staining using a-bromodeoxyuridine monoclonal antibody diluted 1:8 as a primary antibody (Becton Dickinson, San Jose, CA, USA). The cells were then analyzed by flow cytometry (FACSCalibur, Becton Dickinson). To analyze the DNA content, cells were fixed with cold 70% ethanol, treated with 0.5 mg/mL RNAse, and stained with 20 μg/mL propidium iodide.

### Cell motility assay

The motility assay was conducted using Transwell chambers (Costar, Cambridge, MA, USA) with an 8-μm pore size, polyvinylpyrrolidone-free, polycarbonate filters (Nucleopore, Pleasanton, CA, USA). IMDM plus 10% FBS was placed in the lower compartment of the chamber. MG-63 and U-2OS OS cells (10^5^) were re-suspended in IMDM plus 10% FBS with or without NT157 (range 1–3 μM) and then seeded in the upper compartment. The chambers were incubated at 37°C in a humidified atmosphere containing 5% CO_2_ for 18 h. The cells that migrated toward the filter to reach the lower chamber base were counted after Giemsa staining. All experiments were performed in triplicate.

### Wound-healing assay

The cell motility was also assessed with a wound-healing assay. Briefly, MG-63 and U-2OS OS cells were plated into 60-mm cell culture plates and allowed to grow to confluence in 10% FBS containing IMDM medium. A 1-mm wide scratch was made across the cell layer using a sterile pipette tip. The medium was changed to remove floating or damaged cells. After 5, 8, and 24 h of treatment with or without NT157 (1–3 μM), the cells that had migrated over the denuded area were observed, and pictures were taken at specific time points.

### Western blotting

Cells were treated with NT157 (0.3–1.3 μM) for 48 h or left untreated, and cell lysates were prepared and processed as previously described (30). The membranes were incubated overnight with the following primary antibodies: anti-Shc clone PG-797, anti GAPDH, anti-β-actin (Santa Cruz Biotechnology, San Diego, CA, USA), anti-phospho-Akt (Ser473) clone 736E11, anti-Akt, anti-ERK (Cell Signaling Technology, Beverly, MA, USA), anti-phospho-ERK (Tyr202/Tyr204) (Covance, Princeton, NJ, USA), anti-IRS-1 (Upstate Biotechnology, Temecula, CA, USA), anti-IRS-2 (Abcam, Cambridge, UK), and phospho-IRS-1 (Tyr612) (Invitrogen, USA); anti-rabbit or anti-mouse antibodies conjugated to horseradish peroxidase (GE Healthcare, Piscataway, NJ, USA) were used as secondary antibodies.

### Statistical analysis

IC_50_ values were calculated from the linear transformations of the dose–response curves. To define drug–drug interactions (in terms of synergism, additivity, or antagonism), the combination index (CI) of each two-drug treatment was calculated with the isobologram equation (31) using the CalcuSyn software (Biososoft, Ferguson, MO, USA).

## Results

### *In vitro* activity of NT157 in OS cells

**Figure 1:**
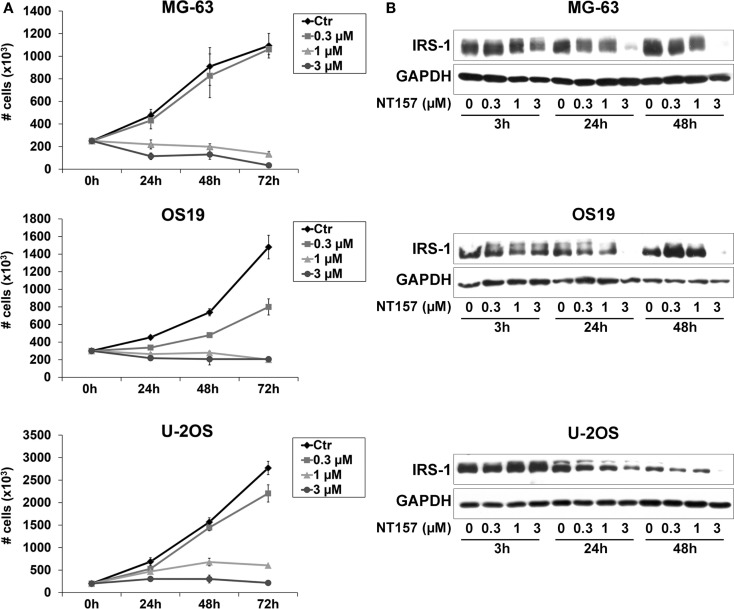
**(A)** The *in vitro* sensitivity to a selected inhibitor of IRS-1/2, NT157, for a panel of OS cell lines. Cell growth was assessed by staining cells with Trypan Blue and counting viable cells after up to 72 h of exposure to NT157 (0.3–3 μM) in MG-63, OS-19, and U-2OS cells. *Points* indicate three independent experiments; *bars* indicate the SE. **(B)** Downregulation of IRS-1 protein level in OS cells in response to NT157. Growing MG-63, OS-19, and U-2OS cells were treated with or without NT157 (0.3–3 μM) for 3–48 h. The expression of IRS-1 was determined by western blotting using 40 μg of total protein cell lysate. GAPDH was used as a loading control. The figure shows data representative of two independent experiments.

We previously demonstrated that the IGF system, including its critical mediator IRS-1, is involved in the regulation of OS cell proliferation (26). Thus, the efficacy of the selective inhibitor of IRS-1/2 NT157 was investigated in three representative OS cell lines. Growing MG-63, OS-19, and U-2OS cells were treated with different concentrations of the compound (0.3–3 μM) for up to 72 h (Figure 1A), and a dose–response proliferation was assessed by Trypan Blue cell counting assay. A strong dose-dependent inhibition of growth was observed in all cell lines tested, showing IC_50_ values at sub-micromolar doses (ranging from 0.3 to 0.8 μM) after 72 h of treatment. NT157 reportedly acts via the downregulation of IRS-1 (27). Our cellular models expressed high basal levels of IRS-1 protein. The exposure of three OS cell lines to NT157 elicited dose- and time-dependent decreases in the IRS-1 protein levels. The maximal activity was reached already after 24 h of treatment (Figure 1B), confirming that the inhibitory effects on tumor growth in OS were related to destruction of IRS-1.

### NT157 efficiently affects migration ability of MG-63 and U-2OS OS cells

**Figure 2:**
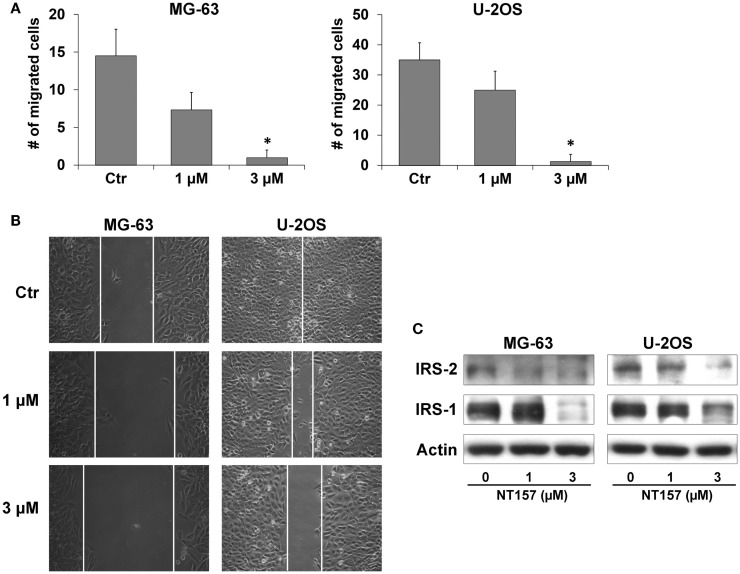
**NT157 inhibits migration ability of OS cell lines**. **(A)** Cell migration of MG-63 and U-2OS cells after treatment with NT157 for 18 h. Columns show the mean of three independent experiments: bars indicate the SE. *p < 0.05, Student’s *t*-test. **(B)** Wound-healing assay in MG-63 and U-2OS cells. Representative pictures were taken after 24 h of treatment with NT157 (1–3 μM). Magnification 100×. **(C)** Inhibition of cell motility is mediated by downregulation of IRS-2 and IRS-1 in MG-63 and U-2OS cells after 24 h of treatment with NT157 (1–3 μM). β-actin was used as loading control.

Because OS is a highly metastatic tumor, the effect of NT157 on cellular migration was also evaluated. MG-63 and U-2OS cells were pre-incubated with inhibitor (1–3 μM) for 24 h, and a motility assay was performed using Transwell chambers. Both cell lines displayed a significant reduction in motility (*p* < 0.05) compared with the control in response to 3 μM NT157 (Figure 2A); this effect was also confirmed with a wound-healing assay (Figure 2B). This effect could be attributed to the inhibition of IRS-2 protein (27), which is known to be essential for tumor metastasis (32), by NT157 (Figure 2C).

### NT157 treatment induces cell cycle arrest and inhibits IGF system signaling

**Figure 3:**
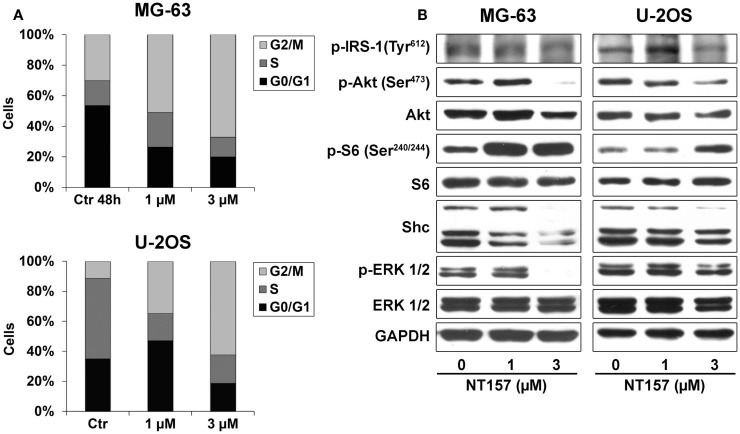
**(A)** Analysis of NT157 effects on cell cycle after 48 h of treatment (1–3 μM) in MG-63 and U-2OS OS cell lines. Columns show the mean percentage of cells in different cell cycle phases as measured by flow cytometry. **(B)** Analysis of major downstream signaling of IRS-1 after treatment with or without NT157 (1–3 μM) by western blotting using 40 μg of total protein cell lysate. GAPDH was used as a loading control. The figure shows data representative of two independent experiments.

The effect on proliferation was related to a modification of the cellular content. Treatment with 1–3 μM NT157 for 48 h substantially increased the percentage of cells in the G2/M phase (Figure 3A), which inhibited cell cycle progression, in keeping with observed in other cancer cell lines (28). In addition, the manual counting of viable cells revealed that NT157 significantly decreased the number of viable cells without inducing apoptotic cell death (data not shown). This finding demonstrated the cytostatic rather than cytotoxic activity of NT157 in this tumor histotype.

To assess if the effect on cell cycle was mediated by the inhibition of principal pathways downstream of IRS-1, cells were treated with different concentrations of NT157 for 48 h. A dose-dependent experiment showed that NT157 could downregulate the Tyr-phosphorylation of IRS-1 and, in turn, phosphorylation of Akt and ERK in the MG-63 and U-2OS cell lines (Figure 3B). The shc protein level was only weakly downregulated in MG-63 cells and remained unchanged in U-2OS cells, suggesting that the inhibition of IRS-1 by NT157 did not elicit significant compensatory effects on other signaling adaptors. Interestingly, blocking the PI-3K/Akt pathways resulted in significant S6K phosphorylation following treatment with the IRS-1 inhibitor. A number of works showed that p70^S6K^, which is downstream of mTOR, could phosphorylate serine residues on IRS-1 to result in proteasomal degradation (33, 34). Because NT compounds induce strong Ser-phosphorylation of the IRS protein, their inhibitory activity could be amplified by feedback that involves p70^S6K^.

### NT157 effects in combination therapy

Several studies indicated that the pharmacologic inhibition of mTOR, which leads to the downregulation of IRS-1, results in the compensatory upregulation of AKT activity via increased levels of IGF-1R and IRS-1 (35). Thus, NT157 administration in association with therapies that target mTOR may be advantageous, which agrees with our findings. In particular, the combination of Everolimus and NVP-BEZ235 has been studied in OS cell lines. Everolimus is an mTOR inhibitor, while NVP-BEZ235 is a dual inhibitor of PI-3K/mTOR signaling. Both drugs have been reported to be active in OS models (36, 37). Simultaneous treatments were made in association with chemotherapeutic agents in a fixed ratio for 72 h and cell viability was determined by MTT assay. Synergistic or addictive effects with respect to single agents are expressed as the CI [Synergism: CI <0.9; additive: 0.9 ≤ CI ≥ 1.10 according to Chou et al. (31)]. The effectiveness of Everolimus and NVP-BEZ235 significantly increased in the combination regimens compared to treatment with these drugs alone, demonstrating a synergistic effect [synergism CI ≤ 0.9 (Table 1)]. Conversely, combined treatment with methotrexate, cisplatin, and doxorubicin, the main drugs used to treat sarcoma patients, showed that NT157 produced only modest additive effects, except for the association with doxorubicin in MG-63 cells, which also demonstrated a synergistic increase in efficacy (Table 1).

**Table 1:** ***In vitro* combination study of NT157 with conventional and targeted drugs in OS cells**.

Drug combination	MG-63		U-2OS	
	CI ± SE	Effects	CI ± SE	Effects
NT157 + Everolimus	0.66 ± 0.2	Synergistic	0.47 ± 0.08	Synergistic
NT157 + NVP-BEZ235	0.59 ± 0.06	Synergistic	0.82 ± 0.02	Synergistic
NT157 + Doxorubicin	0.79 ± 0.06	Synergistic	1.55 ± 0.7	Additive
NT157 + CDDP	0.92 ± 0.08	Sub-additive	1.22 ± 0.05	Additive
NT157 + MTX	1.11 ± 0.05	Additive	>100	Additive

**Figure 4:**
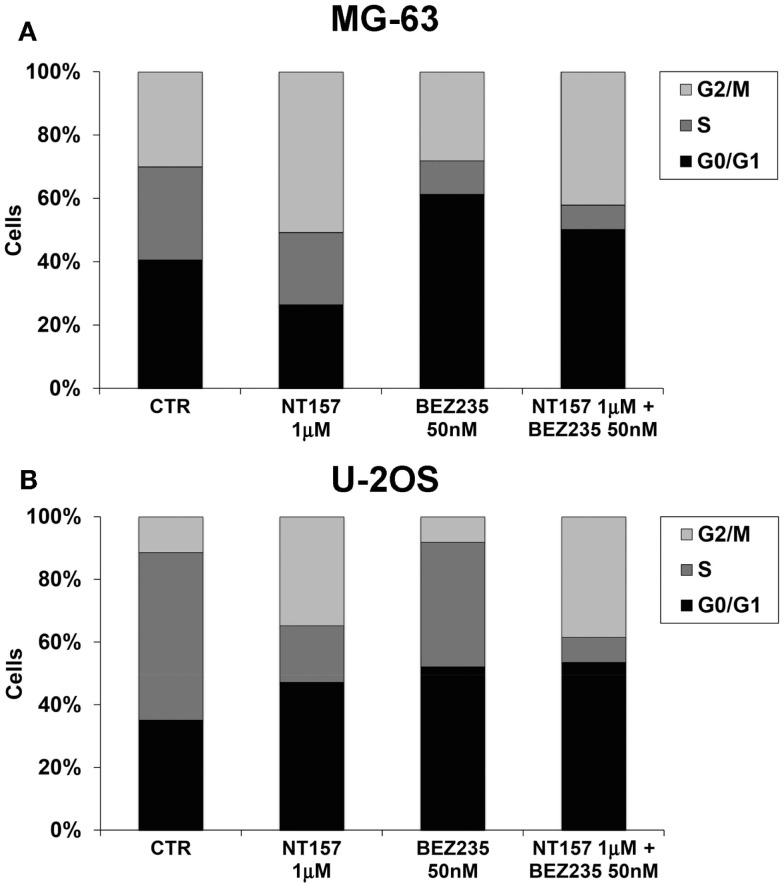
**Inhibition MG-63 (A) and U-2OS (B) cell cycle by NT157 (1 μM) in association with PI-3K/mTOR inhibitor NVP-BEZ235 (50 nM)**. Columns show the mean percentage of cells in different cell cycle phases as measured by flow cytometry.

The advantageous effect of the NT157 and NVP-BEZ235 combination treatment was also confirmed with a cell cycle analysis. Both the percentages of G2/M phase cells and G0/G1 cells increased in response to NT157/NVP-BEZ235 combination treatment in the MG-63 and U-2OS cell lines (Figure 4).

## Discussion

The prognosis of patients with recurrent and metastatic OS remains poor. Thus, developing new strategies to block pathways that are essential for tumor growth and metastasis provide possible alternatives to improve the outcomes for these patients. IGF signaling is a central player in the induction/maintenance of the epithelial mesenchymal transition (EMT) and cell stemness, two strictly related programs that play a key role in metastatic spread and resistance to cancer treatments (2). Accumulating evidence has indicated that the IGF-1 signaling pathway is dysregulated in OS (38). A recent study of genome-wide gene expression and subsequent gene set analysis in OS cell lines and biopsies demonstrated increased IGF signaling in high-grade OS compared with OS progenitors (39). Several reports demonstrated that the expression levels of adaptor protein IRSs, which signaling from upstream activators, like IGF-IR and IR, to multiple downstream effectors to modulate normal growth, metabolism, survival, and differentiation (4), are increased and hyper-activated in many human tumors (40). In OS, altered IRS-1 expression inhibits osteoblastic differentiation and enhances tumor malignancy (26).

In this study, we reported the preclinical antitumor activity of NT157, a selective inhibitor of IRS-1/IRS-2 in OS. The *in vitro* and *in vivo* efficacy of NT157 was recently reported in several tumors (27, 28). Treatment with NT compounds *in vivo* significantly inhibited the growth of vemurafenib-resistant melanoma and displayed potent antitumor effects in ovarian and prostate cancer (28). In particular, in androgen-dependent and -independent prostate carcinoma, NT157 decreases the expression of IRS proteins and downregulates IGF-1R-mediated AKT activation, leading to cell cycle arrest, apoptosis, and a delay of castrate-resistant prostate cancer progression in xenografts (28). We have demonstrated that *in vitro* NT157 can inhibit proliferation, cell cycle progression, and motility in different OS cell lines. Importantly, short-term exposure to NT compounds has been demonstrated to be sufficient to gain long-lasting antitumoral effects (27). From a clinical point of view, this attribute allows for treatment relatively infrequent treatments, which should reduce side effects.

Potential inhibitors of the PI-3K–AKT–mTOR pathway, which is frequently dysregulated in cancer (41), are expected to have therapeutic utility in many tumors, and several of these inhibitors are under current investigation as therapeutic agents for cancer (42). In the last decade, particular attention in sarcoma treatment has been focused on the blockade of mTOR by rapamycin and derivatives, which were reported to inhibit the growth of OS cells lines *in vitro* and in xenografts (43–46). However, a recent phase I study of pediatric solid cancer demonstrated no objective response to temsirolimus, an analog of rapamycin, in OS patients (47). This discrepancy between preclinical and clinical results is explained by the presence of negative feedback that activates IGF-IR downstream signaling and protects against mTOR inhibition (43). The inhibition of mTOR results in the hyperphosphorylation and activation of Akt, leading to resistance to apoptosis and increased cell growth. This effect is abrogated by the inhibition of IGF-1R (43). *In vitro* and *in vivo* studies showed that combined anti-IGF-1R antibody and mTOR inhibitor treatment decreased pAKT activation (24, 43, 48, 49). Based on these findings, recent clinical trials showed that this combination was effective in patients with sarcomas (50, 51). Here, we have demonstrated that NT157 exhibits a strong synergistic effect in OS cells when combined with Everolimus, an orally administered rapamycin analog, suggesting that combination therapy based on mTOR and IRS-1 inhibitors may be an appropriate strategy to enhance mTOR-targeted anticancer therapy in this tumor. Pignochino et al. recently reported that the combination of Everolimus and sorafenib, a multikinase inhibitor, inhibited OS cell lines (52), showing that the mTORC2 upregulation observed in sorafenib-treated OS may represent the escape mechanism from this targeted therapy. Combining sorafenib with the mTOR inhibitor Everolimus, fully blocked both mTOR complex 1 (mTORC1) and mTOR complex 2 (mTORC2), which enhanced the antitumor, antimetastatic, and antiangiogenic activities of this treatment. Although the combination of sorafenib and Everolimus was efficacious for patients with advanced or unresectable OS, a phase 2 clinical trial showed that 45% of patients were free from progression at 6 months, suggesting that this strategy should be further studied, either by modulating the same drugs or improving the inhibitory specificity with novel targeted therapies (53). In this context, combined treatment of NT157 with rapamycin analogs will represent an advantageous therapeutic alternative for OS patients. Interestingly, our combination experiments have also demonstrated that NT157 exerted synergistic effect with NVP-BEZ235, a dual class I PI-3K/mTOR inhibitor (54). NVP-BEZ235 has shown promising therapeutic activity in carcinomas (55, 56) and lymphomas (57). Several reports demonstrated its effectiveness for the treatment of bone sarcoma and more specifically, for OS (36, 58, 59). Manara et al. (36) reported that the combination of NVP-BEZ235 with the TK inhibitor NVP-AEW541 synergistically affected the U-2OS cell line. Combined with these findings, our results further support that the combination of IRS-1/2 inhibitor NT157 with NVP-BEZ235 is applicable for the treatment of OS.

The NT-mediated suppression of IRS-1 and IRS-2 has an important clinical implication for overcoming drug resistance. The inhibition of IRS proteins following NT157 treatment clearly improves the response of prostate cancer xenografts to docetaxel (28). In addition, recent studies (27, 60) demonstrated that acquired resistance to the B-RAFV600E/K inhibitor in melanoma is mediated by increased levels of IGF-1R and IRS-1, and this resistance can be effectively reversed by treatment with NT157. Other targeted therapies specifically block IGF-1R and induce a compensatory activation of IR via IGF-II, which leads to drug resistance (61–63). In this context, NT157 disrupts signaling downstream of both IGF-1R and IR and reduces the probability of drug resistance. Finally, short-term exposure to NT compounds has been demonstrated to be sufficient to gain long-lasting antitumoral effects. From a clinical point of view, this attribute allows for relatively infrequent treatments, which should lead to reduced side effects.

Overall, our data provide evidence that the docking protein IRS-1 is a potential target for treating OS. Due to the lack of apoptotic activity, NT157 is a promising adjuvant drug for bone sarcomas. These results suggest the need for future testing of the combination therapy of mTOR inhibitors and NT compounds in a clinical setting for the treatment of patients with chemorefractory, advanced OS.

## Author Contributions

Conception and design: CG, KS. Acquisition, analysis, or interpretation of data: CG, MC, CM. Writing, review, and/or revision of the manuscript: CG, HR, PP, KS. Final approval of the version to be published: KS, PP.

## Conflict of Interest Statement

Dr. Hadas Reuveni has a patent entitled “Novel protein kinase modulators and therapeutic uses thereof” with royalties paid to TyrNovo, and a patent entitled “Combinations of insulin receptor substrate modulators and protein kinase modulators for treating cancer” with royalties paid. The other co-authors declare that the research was conducted in the absence of any commercial or financial relationships that could be construed as a potential conflict of interest. The Specialty Chief Editor Antonino Belfiore declares that, despite having collaborated with author Cecilia Garofalo, the review process was handled objectively and no conflict of interest exists.
